# The Relationship between Reactive Balance Control and Back and Hamstring Strength in Physiotherapists with Non-Specific Back Pain: Protocol for a Cross-Sectional Study

**DOI:** 10.3390/ijerph18115578

**Published:** 2021-05-23

**Authors:** Erika Zemková, Eva Ďurinová, Andrej Džubera, Henrieta Horníková, Juraj Chochol, Jana Koišová, Michaela Šimonová, Ludmila Zapletalová

**Affiliations:** 1 Department of Biological and Medical Sciences, Faculty of Physical Education and Sports, Comenius University in Bratislava, 814 69 Bratislava, Slovakia; henrieta.hornikova@uniba.sk; 2 Sports Technology Institute, Faculty of Electrical Engineering and Information Technology, Slovak University of Technology, 812 19 Bratislava, Slovakia; 3 Faculty of Health Sciences, University of Ss. Cyril and Methodius in Trnava, 917 01 Trnava, Slovakia; eva.durinova@ucm.sk (E.Ď.); jana.koisova@ucm.sk (J.K.); michaela.simonova@ucm.sk (M.Š.); zapletalovalidi@gmail.com (L.Z.); 4 Department of Neurosurgery, Slovak Medical University and University Hospital—St. Michael’s Hospital, 811 08 Bratislava, Slovakia; andrej.dzubera@nsmas.sk (A.D.); juraj.chochol@nsmas.sk (J.C.)

**Keywords:** back problems, Low Back Pain Scale, maximum voluntary isometric contraction, postural stability, unexpected external postural perturbations

## Abstract

Back pain is one of the most costly disorders among the worldwide working population. Within that population, healthcare workers are at a high risk of back pain. Though they often demonstrate awkward postures and impaired balance in comparison with healthy workers, there is no clear relationship between compensatory postural responses to unpredictable stimuli and the strength of related muscle groups, in particular in individuals with mild to moderate back pain. This paper presents a study protocol that aims to evaluate the relationship between peak anterior to peak posterior displacements of the center of pressure (CoP) and corresponding time from peak anterior to peak posterior displacements of the CoP after sudden external perturbations and peak force during a maximum voluntary isometric contraction of the back and hamstring muscles in physiotherapists with non-specific back pain in its early stages. Participants will complete the Oswestry Disability Questionnaire. Those that rate their back pain on the 0–10 Low Back Pain Scale in the ranges 1–3 (mild pain) and 4–6 (moderate pain) will be considered. They will undergo a perturbation-based balance test and a test of the maximal isometric strength of back muscles and hip extensors. We assume that by adding tests of reactive balance and strength of related muscle groups in the functional testing of physiotherapists, we would be able to identify back problems earlier and more efficiently and therefore address them well before chronic back disorders occur.

## 1. Introduction

In 2019, the WHO reported that among musculoskeletal conditions, low back pain (LBP) is the leading cause of disability worldwide. In particular, work-related back pain is a major cause of reduced productivity and increased disability of workers, which places a significant financial burden on healthcare systems. Of all the professions, healthcare workers are at the highest risk of back problems [1,2,3,4,5,6,7,8,9,10]. This is due to overexertion of the back while handling patients, which may lead to awkward spinal postures and impaired static and dynamic balance.

Poor balance in itself may contribute to more severe back pain. Indeed, most LBP patients demonstrate impaired postural stability. For instance, differences exist in postural control strategy, the CoP displacement, and muscle activation patterns between people with and without non-specific LBP [11]. A greater postural instability, denoted by higher CoP velocity and excursions, has been found in people with non-specific LBP compared to healthy controls [12]. Their impaired postural stability seems to be associated with the presence of pain, whereas it is not related to the exact location and pain duration. There is no relationship between the magnitude of CoP excursions and the pain intensity.

Therefore, there is a need to identify more appropriate variables associated with back pain and to specify test conditions tailored to the requirements for assessment of healthcare workers with non-specific back pain in its early stages. Both the CoP and center of mass (CoM) variables should be measured in dynamic conditions with higher task demands. Standing on a spring-supported platform or a foam surface during testing is more efficient in identifying within- and between-group differences when compared to tests of static balance [13]. A good discriminatory accuracy in differentiating between sedentary and physically active young adults was also reported during a perturbation-based balance tests [14]. On the other hand, there were no significant differences in the magnitudes of CoP and CoM displacements in the mediolateral direction during unexpected surface translation in individuals with chronic LBP and healthy controls [15]. However, those with chronic LBP showed an earlier peak CoM displacement and later onset of initial CoP displacement [15]. Nonetheless, readjustment of postural stability in the anteroposterior direction after perturbations elicited by weight unloading has yet to be investigated.

The lumbar extensors and hip musculature may be an important factor in determining the motor control dysfunctions, such as limited balance, that arise in chronic LBP [16]. Lumbar extension strength has been shown to correlated significantly with Star Excursion Balance Test scores and explained ~19.3% to ~37.8% of its variance in the chronic LBP group and ~9.5% to ~16.9% in the asymptomatic group [16].

Traditionally, good isometric endurance of back muscles was sought to prevent first-time LBP occurrence [17]. The Sørensen test has been frequently used [18]. Such a measurement has been recommended as a standard for lifting tasks [19]. This was based on evidence that lower isometric strength is associated with LBP. However, the risk of back problems increases threefold when the requirements for lifting tasks are beyond or equal to their strength capacity. Static strength measurements underestimate the loads on the spine under dynamic conditions. The predicted spinal loads are 33–60% less under static than dynamic conditions depending on the lifting technique [20]. The recruitment patterns of trunk muscles and thus the internal loading of the spine are also different under these two conditions. Therefore, tests performed under dynamic conditions seem to be more appropriate for healthy as well as LBP populations. An exercise in the form of a deadlift to high pull that involves the major muscle groups in the lower and upper body may be able to simulate lifting tasks [21]. In such a case, a predictor of lifting performance with light loads might be a peak rate of force development (RFD) produced during a maximum voluntary isometric contraction (MVC) of the back muscles [22]. Thus, besides MVC peak force generated by back muscles, the subjects’ ability to produce a maximum force in a short period of time should by determined to obtain further insight into the loaded lifting performance in those prone to LBP.

In comparison with these frequently used strength and endurance tests of back muscles, less attention has been paid to the assessment of hamstring strength in LBP individuals and the associations with variables of postural and core stability measured in more challenging conditions. Though there is the belief that an association exists between a stronger body core and shorter reaction time, or stronger lower limbs and faster recovery, there is also an opposite view that suggests that stronger lower limbs contribute to slower recovery [23]. Therefore, a question remains as to whether reactive balance control after unpredictable stimuli is associated with the strength of relevant muscle groups in the high-risk population of healthcare workers.

This paper presents a protocol for a cross-sectional study that will investigate the relationship between peak anterior to peak posterior displacements of the CoP and corresponding time from peak anterior to peak posterior displacements of the CoP after sudden external perturbations and peak force during MVCs of the back and hamstring muscles in physiotherapists with non-specific back pain, in particular those with mild to moderate back pain. We will also investigate how the ability to produce maximum force in a short period of time during MVCs of the back and hamstring muscles relates to postural responses to externally induced perturbations in a control group without back pain.

## 2. Materials and Methods

### 2.1. Study Design

**Figure 1:**
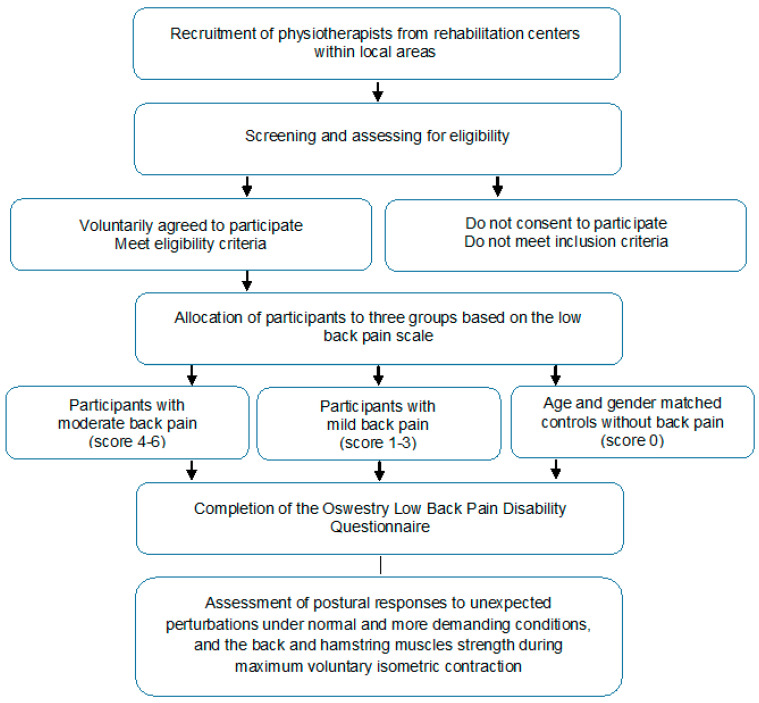
Flowchart of the study design.

This cross-sectional study is designed to evaluate associations among measures of a perturbation-based balance test and back and hamstring strength tests in physiotherapists with non-specific back pain. The study will be implemented and reported in line with the Standard Protocol Items: Recommendations for Interventional Trials (SPIRIT) statement. The study design is illustrated in Figure 1.

### 2.2. Participants and Setting

A group of 82 female and male physiotherapists with non-specific back pain will be recruited from rehabilitation centers within local areas. The control group will include 41 participants. These effect sizes are sufficient to identify significant interaction effects. The contact with these centers and the necessary support for recruitment of subjects and data collection has been will be provided. We expect approximately 90% of eligible physiotherapists to consent to participate in the study. The timetable will be specified when the COVID-19 pandemic is over.

### 2.3. Inclusion and Exclusion Criteria

Subjects will be screened for eligibility by the research team members. Those who report non-specific back pain [24,25] for more than 12 weeks [26,27,28,29] will be eligible. Inclusion criteria for LBP individuals and healthy controls will require no history of orthopedic or neurological conditions that could influence balance. Participants scoring ˂7 out of 10 on a numeric pain rating scale will be eligible for inclusion. Those who have previously undergone medically invasive procedures for back pain or have a diagnosis that may explain their low back symptoms (e.g., former injury or illness within the last 12 months) will be excluded. Exclusion criteria will also include pain not primarily generated from the musculoskeletal system, infections, diabetes, and pregnancy. As the strength of hamstring muscles will also be evaluated, participants who will report any other current muscular, joint, or neurological conditions affecting lower limb function will be excluded.

### 2.4. Allocation

Participants will be divided into two groups according to the Numeric Rating Scale (mean of three assessments: current LBP, the usual/mean LBP within the last two weeks, and the worst LBP within the last two weeks), which is widely used in medical settings to obtain information about the level of a patient’s pain. The scale ranges from 0 (no pain at all) to 10 (unbearable pain). Participants experiencing mild pain (pain score 1–3), which is easy to manage physically as well as psychologically and does not interfere with most daily activities, and moderate pain (pain score 4–6), which interferes with daily activities and requires changes to manage pain symptoms during the last three months, will be considered. A control group of matched age, gender, and sample size will include those reporting no back pain.

### 2.5. Sample Size Estimation

The statistical power was calculated using the software program G*power 3.1 for Mac OS X. The calculation of the sample size was conducted with α = 0.05 (5% change of type I error) and 1 − β = 0.80 (power 80%) and using the results from our previous measurements. This indicated that a sample size of 34 individuals per group is needed to identify significant interaction effects. To reach this target sample size and achieve sufficient participant enrolment, 20% will be added to allow for dropouts.

### 2.6. Procedures

Participants will be asked to avoid exercises of higher intensity prior to the investigation. Before assessment, they will be given information on each testing protocol and the instructions during measurements. To eliminate learning effects, they will be required to practice a whole procedure beforehand. Tests will be conducted by the same examiners at the same time of the day for all participants. In comparison with frequently used laboratory tests, the use of portable and user-friendly diagnostic systems suited for testing in field conditions will be preferred in the present study. Using easy to administer tests that can reveal in a relatively short time period the impairment of postural and core stability and reduction in strength of particular muscle groups in LBP individuals could increase their applications in practice.

### 2.7. Data Collection and Management

The study will comprise the collection of personal demographic and outcome data. The questionnaire data will be transferred to a database and checked for correctness by research team members to ensure their quality. All information and outcome data will be stored in password-protected computers, which will be accessible by authorized research team members. Data management during the project and after the project’s completion will provide information on how data will be collected, documented, stored, and archived.

### 2.8. Descriptive Measures

Upon arrival at the laboratory, the participants’ characteristics will be summarized. These will include age, height, body mass, body composition, gender, and additional information related to back problems such as the amount of daily practice with clients, the type and duration of sporting activities, previous injuries, diseases, and so forth.

### 2.9. Primary Outcomes

#### Oswestry Disability Index

Participants will complete the Oswestry Low Back Pain Disability Questionnaire, which gives a subjective score of the level of function and/or disability in daily activities [30]. It is considered the ‘gold standard’ of low back functional outcome tools [30]. The Oswestry Disability Questionnaire data are reliable and have a scale of adequate width to reliably reveal worsening or improvement in most subjects [31]. The questionnaire consists of two parts—Oswestry Disability Index (ODI) version 2 and Visual Analogue Scale (VAS). The ODI questionnaire is designed to give information about how back or leg pain affects a patient’s ability to manage in everyday life. The ODI consists of 10 questions with a single choice of 6 answers. The VAS is a measurement instrument to measure the strength of pain experienced by a patient. Using a ruler, the score is determined by measuring the distance (mm) on a 10 cm line between ‘no pain at all’ and ‘my pain is as bad as it could be’. The patient marks a score from 0–10. A higher score indicates greater pain intensity. The pain VAS is a unidimensional measure of pain intensity, widely used in diverse adult populations. The combination of ODI and VAS is accepted worldwide and used in clinical practice because it is easy to use among patients and physicians.

### 2.10. Secondary Outcomes

#### 2.10.1. Postural Responses to Unexpected Perturbations

Participants will perform a perturbation-based balance test. They will be instructed to perform the test under two conditions: (1) bipedal and (2) tandem stance. Participants’ eyes will be focused on a spot on the wall at their eye level. They will be asked to stand barefoot on a force plate, a shoulder width apart, while their arms will be held in front horizontally. They will be asked to hold a bar with a 2 kg fixed load in their hands. After the test initiation, a signal from the computer will trigger a random load release over a 5 s period, therefore the participant will receive no cues as to when the perturbation will occur. The load release will produce a sudden change in the external forces acting on the participant that will lead to a slight anterior and a greater posterior CoP displacement. This postural perturbation will cause only a sway response, so the participant will not need to perform a step to maintain balance. The perturbation will be determined by peak anterior and peak posterior displacements within 1 s following the load drop. The recording will end 2–3 s following the load drop.

Three trials of each test condition will be randomly performed. The primary outcome measures will include peak anterior to peak posterior displacement of the CoP and the time from peak anterior to peak posterior displacement of the CoP. Secondary measures will include peak anterior displacement of the CoP, peak posterior displacement of the CoP, the time to peak anterior displacement of the CoP, and the time to peak posterior displacement of the CoP. These variables will be monitored by means of the FiTRO Sway Check (FiTRONiC, SVK). This system registers the actual force in the corners of the force plate and calculates an instant CoP position (sampling rate of 100 Hz, 12 bit AD signal conversion, resolution of the CoP position less than 0.1 mm, measuring range of 0–1000 N/s, non-linearity of +/−0.02% FS, combined error of 0.03%, sensitivity of 2 mV/V +/− 0.25%, overload capacity of 150%/sensor). Additionally, a ratio of the CoP anterior to the CoP posterior values will be estimated. The reliability of parameters of the perturbation-based balance test is good to excellent, with low SEM (7.1–10.7%) and high ICC values (0.78–0.92) [16]. This test is also sensitive in discriminating between sedentary and physically active young and early to late middle-aged adults. Good discriminatory accuracy of these variables is indicated by an area under the ROC curve >0.80 [14].

#### 2.10.2. Maximal Strength of the Back and Hamstring Muscles

Before the test begins, the participants will be warmed up by performing two submaximal isometric contractions so as to become accustomed to the procedure. Afterwards, they will be placed into the proper position with knee and hip angles of 141° and 124°, respectively, set up by a handheld goniometer. This position corresponds to the portion of the clean lift during which the highest values of power are achieved [32]. A handlebar of this device will be attached to a floor-mounted load cell. Its height above the floor will be determined for each participant during a familiarization trial. Once the participants are in position, they will initiate the contraction after a countdown of “3, 2, 1, pull”. They will perform three maximal MVCs as forcefully as possible for at least 5 s. Participants will be provided with verbal encouragement at each trial. A minimum of a 2 min rest period will be given between MVC efforts. The visual feedback on the instantaneous force will be provided in real time on a monitor positioned in front of the research team member. Force will be measured using the FiTRO Back Dynamometer (FiTRONiC, SVK). Analog signals will be AD converted and sampled at a rate of 1000 Hz. Peak force will be analyzed.

The same system will be used for measurement of the hamstring strength. Participants, after a warm-up, will perform three 5 s isometric contractions with maximal effort on each leg at 90° of knee flexion while lying on the rehabilitation bed in the prone position. This knee flexion angle is based on findings that showed high reliability of isometric posterior lower limb muscle force [33]. In a randomized design, the left and right leg peak force will be registered. The best result from the three attempts will be taken for the analysis.

The control group without back pain will undergo the same procedures, however, these participants will also perform three MVCs as quickly and as forcefully as possible for at least 3 s. In this case, the peak RFD in addition to peak force during MVCs of the back and hamstring muscles will be analyzed.

Assessment of postural control after sudden external perturbations and maximal isometric strength of the back and hamstring muscles in participants will be completed by the measurement of their low back and hamstring flexibility.

### 2.11. Patient and Public Involvement

The public and patients will be not directly involved in the present study. Local medical centers will provide support for recruitment of physiotherapists with non-specific back pain. Test results will be provided to participants on request and the overall outcomes will be available to them on completion of the study.

### 2.12. Ethics and Dissemination

The procedures described are in accordance with the ethical standards as laid down by the 1964 Helsinki Declaration and its later amendments. Participants will be verbally informed of the main study objective, procedures, risks and benefits, confidentiality, and the voluntary nature of their participation and provided an opportunity to ask questions. Prior to inclusion, written informed consent will be obtained. Projects were approved by the ethics committee of the Faculty of Physical Education and Sports, Comenius University in Bratislava (Nos. 4/2017 and 1/2020).

The findings obtained will be publicly available in particular journals. Research results will also be presented at scientific conferences and disseminated outside and/or within related healthcare centers and/or universities.

### 2.13. Statistical Analysis

Statistical analysis of the data obtained will be conducted using the SPSS program for Windows, version 24.0 (SPSS, Inc., Chicago, IL, USA). The normality hypothesis will be tested using the Kolmogorov–Smirnov test. A parametric analysis will be conducted if the data are normally distributed. Associations between variables of the perturbation-based balance test and maximal isometric back and hamstring muscle strength in participants with mild to moderate back pain will be assessed using Pearson’s product moment correlation coefficient (small: r = 0.10–<0.30, medium: r = 0.30–<0.50, large: r = ≥ 0.50) [34]. Similarly, the correlation between RFD produced during MVCs of the back and hamstring muscles and postural responses to externally induced perturbations in the control group will be determined. A standard multiple regression analysis will be performed to investigate which of these variables of the muscle strength could be significant predictors of compensatory postural responses to unpredictable stimuli in individuals without back pain. The amount of variance explained will be determined by the coefficient of determination (r^2^). A multivariable logistic regression model will be used to determine associations of back pain (intensity and duration) with the covariates (age, gender, body mass, and eventually psychological factors and/or medical history of participants). The significance level will be set at α = 5%. Data will be presented as the mean (standard deviation).

## 3. Discussion

Therapists in health care, including physiotherapists, physical therapists, and athletic therapists, are at high risk of developing LBP [1,3,35,36,37]. The occurrence of back problems will likely rise as patients become heavier with increasing obesity. Therefore, more efforts should be directed toward their prediction by regular assessment of physical factors associated with back pain in its early stages. Among them are those associated with awkward spinal postures, impaired static and dynamic balance, and reduced core and lower limb strength. However, conflicting evidence exists on associations among these performance determinants and the occurrence of back problems.

For instance, a significant relationship was demonstrated between the percentage of time spent in awkward postures in the sagittal plane (trunk flexion ≥45°) and in the frontal plane (lateral bend ≥20°) and LBP in hospital nurses [38]. However, a systematic review by Roffey et al. [39] revealed that there is no association between awkward postures and LBP and that there is no temporal relationship. Weak associations and no evidence for other aspects of causality in certain specific subcategories were demonstrated in few studies [39]. Therefore, it is unlikely that awkward occupational postures are independently causative of LBP in the working population [39].

Further, individuals with LBP often exhibit altered responses to sudden perturbations. In particular, delayed trunk muscle responses [40,41,42], decreased amplitudes of muscle activation [40,43], and increased co-contraction [40] are associated with the occurrence of LBP. However, most studies investigating postural responses to perturbations applied to the body’s trunk have been limited to electromyographic (EMG) recordings while less attention has been paid to the assessment of muscle strength and power.

The trunk and hip muscle strength contributes to lumbar spine stability, particularly during functional tasks. Additionally, a low level of the trunk muscle co-contraction is important for the body’s core stability [44]. Such a level of stiffness provides necessary stability against minor perturbations. In particular, direction-specific muscle reflex responses play an essential role in the stability of the body’s core when encountering unexpected postural perturbations [44].

The evidence has demonstrated that shortening, weakness, and/or muscle stiffness of the pelvic and lumbar regions contribute to LBP. There is an association between decreased stability and an increased risk of low back or knee injury [44]. These individuals demonstrate impaired postural stability, abnormal recruitment patterns of the trunk muscles, and delayed muscle reflex responses to unexpected trunk unloading [44]. Additionally, lower strength of knee extensors and hip abductor/extensors has been reported in LBP patients than healthy controls [45,46,47,48,49]. Provided that the muscles of lower limbs, particularly the hip muscles, play an important role in the stability of the lumbar spine [50], it is likely that lumbar instability and inefficient lumbopelvic motor control, together with weakness in hip muscles, may contribute to LBP development. However, a relationship between hip strength and back pain has not been found [51,52].

Thus far, the association of hip weakness with the presence of non-specific LBP has not been sufficiently investigated. Although several studies have demonstrated the efficiency of trunk and lower limb strengthening exercises for improvement of core stability [53,54,55], less attention has been paid to core stability in relation to the strength of particular muscle groups. This is due to a paucity of easy to administer tests using portable and user-friendly diagnostic systems suited for testing in field conditions.

Our study will address this gap in the available research. It will provide insight into the association between the ability of subjects to maintain postural stability after an unexpected perturbation and maximal isometric strength of the back and hamstring muscles. Our attention will be paid to healthcare workers, namely physiotherapists with mild to moderate non-specific back pain in whom it is hard to reveal slight impairments of the ability to regulate CoM motion using less sophisticated non-laboratory techniques. Identifying the relationship between these factors will help us to design a test battery tailored specifically for this high-risk population using tests that can be performed in field conditions. The limitation will be the sample consisting of mainly female participants due to larger number of women working in the healthcare sector. However, the LBP prevalence rate in this population is high, with the majority of cases occurring after starting work [3,56].

In addressing LBP prevention, the ability to produce maximum force in a short period of time during MVCs of the back and hamstring muscles will be assessed in the control group without back pain. This method could provide feedback about whether those with higher peak RFD during MVC of relevant muscle groups are capable of responding effectively to unexpected postural perturbations. It can be implemented in healthy subjects who may benefit from such testing by predicting their LBP risk. Revealing impaired postural and/or core stability and reduced strength of relevant muscle groups could support self-help strategies in the prevention of back problems by the application of hip–trunk stabilization and strengthening exercises in their daily activities. This may contribute to a reduction in chronic back problems and consequently lowering healthcare system costs.
